# Evidence for Photoinduced Insulator-to-Metal transition in *B*-phase vanadium dioxide

**DOI:** 10.1038/srep25538

**Published:** 2016-05-09

**Authors:** James Lourembam, Amar Srivastava, Chan La-o-vorakiat, Liang Cheng, T. Venkatesan, Elbert E. M. Chia

**Affiliations:** 1 Division of Physics and Applied Physics, School of Physical and Mathematical Sciences, Nanyang Technological University, Singapore 637371, Singapore; 2 NUSNNI-Nanocore, National University of Singapore, Singapore 117411, Singapore; 3 Department of Physics, National University of Singapore, Singapore 117542, Singapore; 4 Nanoscience and Nanotechnology Graduate Program, King Mongkut’s University of Technology Thonburi (KMUTT), 10140, Thailand; 5 Faculty of Science, King Mongkut’s University of Technology Thonburi (KMUTT), 10140, Thailand; 6 Theoretical and Computational Science Center (TaCS), Faculty of Science, King Mongkut’s University of Technology Thonburi (KMUTT), 10140, Thailand; 7 Department of Electrical and Computer Engineering, National University of Singapore, Singapore 117576, Singapore; 8 Department of Materials Sciences and Engineering, National University of Singapore, Singapore 117576, Singapore; 9 Department of Integrated Sciences and Engineering, National University of Singapore, Singapore 117456, Singapore

## Abstract

Ultrafast optical studies have been performed on epitaxial films of the novel *B*-phase of vanadium dioxide using temperature-dependent optical pump-probe technique. Signature of temperature-driven metal-to-insulator transition was distinctly observed in the ultrafast dynamics — the insulating phase showed two characteristic electronic relaxation times while the metallic phase showed only one. Beyond a threshold value of the pump fluence, the insulating state collapses into a ‘metallic-like’ phase which can be further subdivided into two regimes according to the lengths of the fast characteristic time. The first regime can be explained by lattice heating due to the optical pump; the other cannot be accounted by simple lattice heating effects alone, and thus offers evidence for a true photoinduced phase transition.

Complex oxides provide a variety of materials platform for studying metal-insulator transitions (MIT)^1^,^2^. From a technological point of view, there are two crucial issues in MIT transitions — the ability to manipulate, and determining the speed of such transitions. Ultrafast optical measurements have been proven to be highly reliable in understanding the timescale and role of photoexcitation in these phase transitions^3^. The study of time-dependent carrier dynamics has helped in understanding the contributions of charge, spin, and lattice degrees of freedom to phase transitions in complex oxides^4^,^5^,^6^,^7^. Recent efforts on time-resolved optical measurements have significantly advanced efforts on optical control of MIT in complex oxides, and have gained a lot of interest particularly owing to its promise of realizing ultra high-speed opto-electronic devices^4^,^8^. In the past, several successful attempts to tune the conductivity by light irradiation were observed in manganites, KMoO_3_, NdNiO_3_, magnetites, etc.^9^,^10^,^11^,^12^,^13^,^14^. Furthermore, it has also been found that photodoping causes the destruction of charge and orbital order leading to modification of magnetic and electronic phases^3^,^15^,^16^,^17^,^18^.

Vanadium dioxide is a complex oxide rich in polymorphism displaying several crystal symmetries that could be stabilized depending on the synthesis conditions^19^. The most popular and extensively studied polymorph of vanadium dioxide, VO_2_(*M*), undergoes a temperature-driven MIT at ~340 K with a concomitant transition in crystal structure^2^. VO_2_(*B*) is also another important polymorph with a rich electronic phase diagram that exhibits a broad insulator to semi-metal transition displaying four orders of resistivity increase as the temperature is lowered from 300 K to 150 K^20^,^21^. The schematic energy band diagrams of VO_2_(*B*) near the Fermi level depicting the metallic and the insulating phases are shown in Fig. 1(a). Similar to VO_2_(*M*), the energy band picture in insulating state of VO_2_(*B*) can be described by the separation of the bonding *d*_||_ band below the Fermi level *E*_F_ and π* orbitals above *E*_F_. In the insulating phase, a band gap of ~0.6 eV opens up^22^. Hard X-ray photoelectron spectroscopy (HAXPES) of VO_2_(*B*) seem to suggest a larger *d*_||_ band splitting in the insulating state of VO_2_(*B*), as compared to VO_2_(*M*), and the π* band also shifts to higher energy with respect to the with respect to *d*_||_ band^23^. X-ray photoelectron spectroscopy study reveals that VO_2_(*B*) is metallic at room temperature, unlike VO_2_(*M*) which has an optical band gap of 0.32 eV^23^. Structurally, VO_2_(*B*) adopts a monoclinic crystal structure *C2/m*(#12) with lattice parameters *a* = 1.203 nm, *b* = 0.3693 nm, *c* = 0.642 nm and *β* = 106.6° with no temperature-dependent structural phase transition^24^,^25^. VO_2_(*B*) can be considered as a layered structure composed of two identical atomic layers, the second layer being shifted by 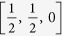 with respect to the first one^25^. VO_2_(*B*) with its deformed VO_6_ octahedra is metastable in nature compared to the rutile phase of VO_2._

While there have been a number of reports on the ultrafast phase transition in VO_2_(*M*) with the pump laser acting as the driving mechanism for non-thermal phase transitions^26^,^27^,^28^,^29^,^30^,^31^,^32^,^33^,^34^,^35^, no time-resolved study on VO_2_(*B*) has been reported yet. Here, we demonstrate that ultrafast spectroscopy can be utilized to identify the electronic phase transition in VO_2_(*B*). We also establish for the first time in VO_2_(*B*) that beyond some critical, optical pump fluence threshold *F*_*th*_, the low-temperature insulating state melts into a ‘metallic-like’ state. *F*_*th*_ is strongly temperature-dependent and there are subtle differences between a light-activated and a thermal-activated phase transition. Single-color pump-probe measurements have been performed in the past to determine the nature of the phase transition in VO_2_(*M*), in terms of both electronic property and atomic structural arrangement^30^.

## Results

**Figure 1:**
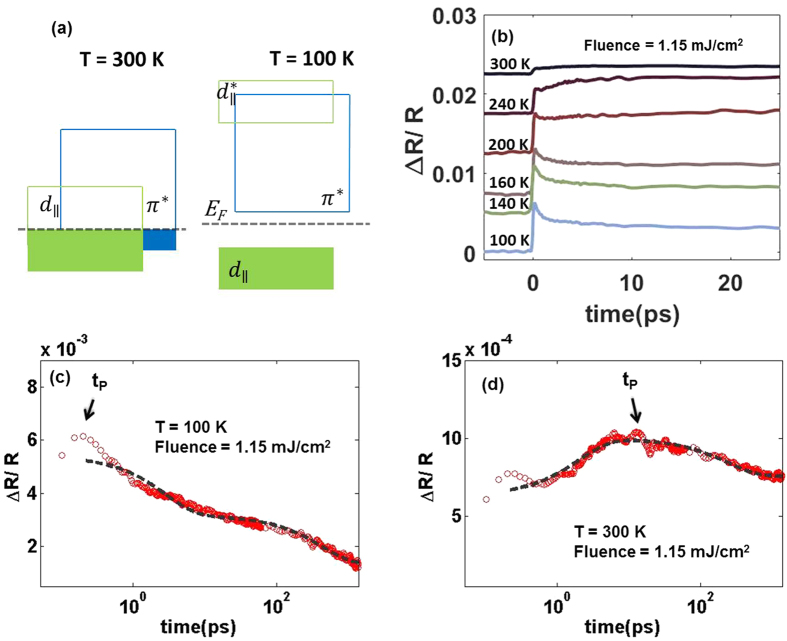
(**a**) Schematic diagram of the VO_2_(*B*) band structure shown along with the Fermi level at the metallic phase (300 K) and the insulating phase (100 K). (**b**) Temperature-dependent time evolution of transient reflectivity Δ*R/R* spectra for VO_2_(*B*) films after photoexcitation by a laser fluence of ~1.15 mJ/cm^2^. Semi-log plot of relative reflectivity fitted with the bi-exponential model for a pump fluence of ~1.15 mJ/cm^2^ at temperatures — (**c**) 100 K and (**d**) 300 K. The arrows represent the delay where the transient reflectivity attains its maximum value, *t*_*P*_.

Time-resolved photoinduced change in reflectivity (or transient reflectivity, Δ*R*/*R*) for all measurements were taken up to 1400 picoseconds (ps). Figure 1(b) shows Δ*R*/*R* of VO_2_(*B*) on SiSTO (Si substrate with SrTiO_3_ as buffer layer) substrate for temperatures between 100 K and 300 K up to 25 ps, for a pump fluence of 1.15 mJ/cm^2^. Between 100 K to 160 K, after an initial positive rise of Δ*R*/*R*, we see an exponential decrease. On the other hand, for *T* ≥ 180 K, Δ*R*/*R* follows a noticeably different trend — after an initial increase, it does not decrease but instead continues to increase, albeit at a slower rate up to several ps, and eventually decreases gradually. These contrasting behaviors in Δ*R*/*R* for *T* ≤ 160 K and *T* ≥ 180 K may suggest the presence of different electronic orders in VO_2_(*B*), which can be better understood by fitting Δ*R*/*R* to an exponential model below.

At a particular temperature, the electronic dynamics of VO_2_(*B*) was fitted to a bi-exponential model described as


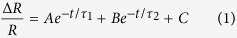


where *A* (*τ*_1_) and *B* (*τ*_2_) represent the amplitudes (relaxation times) of the fast and slow relaxation processes, respectively, while *C* represents the extremely long-lived component. Representative fittings at temperatures 100 K and 300 K are shown in Fig. 1(c,d) respectively — notice that at 100 K Δ*R/R* relaxes immediately after the initial rise after photoexcitation (“relaxation-like”), while at 300 K Δ*R/R* continues to rise after the initial rise (“growth-like”), till ~10 ps before it finally relaxes. The presence of the slow component, *τ*_2_ (300–4000 ps), found in all our measurements, is related to the recovery dynamics. This is consistent with previous time-resolved optical measurements on 50-nm VO_2_(*M*) films, which showed that the characteristic time for recovery from the photoexcited metallic to the insulating phase is ~10 ns^36^. Assuming the recovery dynamics are quite similar, we can expect the photoexcited metallic VO_2_(*B*) to relax into the insulating state before the arrival of the next pump pulse. Also shown in Fig. 1(c,d) is *t*_*P*_ — defined as the time delay where Δ*R/R* reaches a maximum immediately following optical excitation.

**Figure 2:**
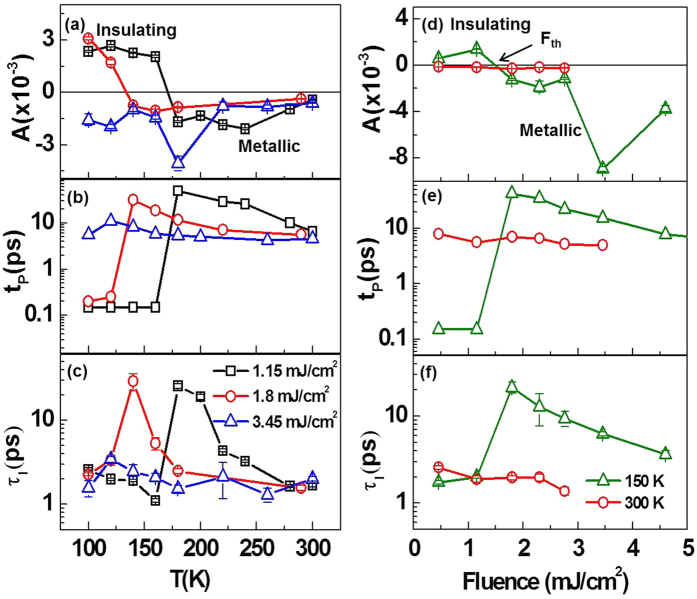
Values of *A, t*_*P*_ and *τ*_1_ for VO_2_(*B*) film as a function of (**a–c**). Temperature, and (**d–f** ). Fluence. *A* and *τ*_1_ were determined from the bi-exponential fitting results, and *t*_*P*_ represents the time delay at the maximum value of Δ*R/R*. The solid black lines in (**a,d**) are references for *A* = 0. The value of *F*_*th*_ at 150 K is indicated by the black arrow in (**d**).

The black data points in Fig. 2(a–c) show the temperature dependence of *A, t*_*P*_, and *τ*_1_, respectively, for a 1.15 mJ/cm^2^ pump fluence. Figure 2(a) clearly shows that *A* changes sign at ~170 K, from positive below 170 K to negative at or above 170 K. Comparing this with temperature-dependent transport and optical conductivity studies on VO_2_(*B*)^20^,^23^, we conclude that the positive *A* corresponds to the insulating state of VO_2_(*B*), while negative *A* corresponds to the metallic state. Figure 2(b) shows that ~180 K also corresponds to the temperature where *t*_*P*_ abruptly increases from sub-ps (in the low-temperature insulating state) to ≥10 ps (in the high-temperature metallic state). Figure 2(c) shows that the fast relaxation time *τ*_1_ is significantly lengthened (to ~30 ps) in the vicinity of 180 K. Note also that *τ*_1_ in the insulating state of VO_2_(*B*), which falls in the range of 1.1–2.8 ps, is comparable to the corresponding values of VO_2_(*M*) determined from transient spectroscopy experiments^27^,^30^.

**Figure 3:**
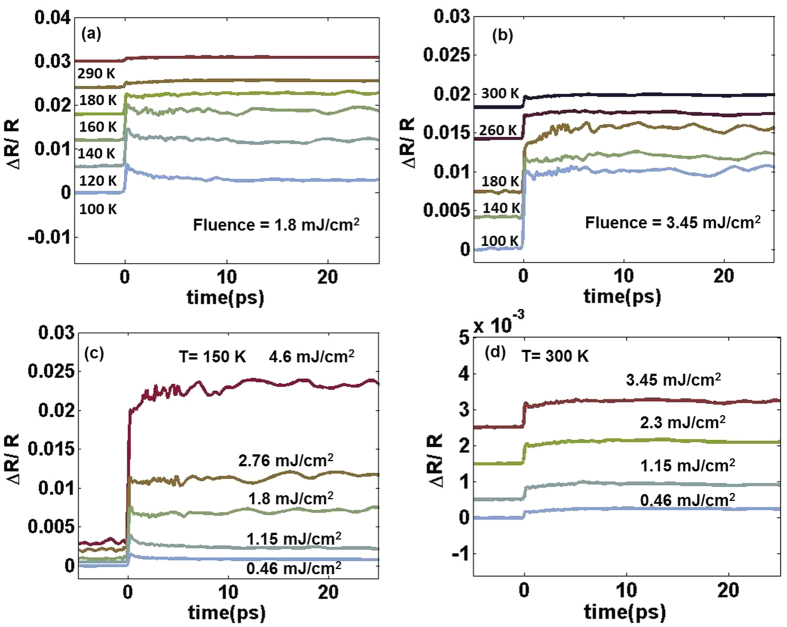
Transient reflectivity at various temperature points for pump fluences (**a**) ~1.8 mJ/cm^2^ and (**b**) ~3.45 mJ/cm^2^ for VO_2_(*B*) thin film. (**c**,**d**) Shows fluence-dependent transient reflectivity at fixed temperatures 150 K and 300 K respectively.

We repeat the measurements at higher excitation fluences. Figure 3(a,b) shows the time dependence of Δ*R/R* at various temperatures for excitation densities 1.8 mJ/cm^2^ and 3.45 mJ/cm^2^ respectively. For 1.8 mJ/cm^2^ pump fluence, the dynamics goes from “relaxation-like” at low temperatures, to “growth-like” at high temperatures — similar to what we see for 1.15 mJ/cm^2^. However, for a 3.45 mJ/cm^2^ pump fluence, we observed “growth-like” dynamics for all temperatures within 25 ps of pump-probe delay.

We now focus on the fluence-dependent Δ*R/R* at a fixed temperature. Figure 3(c) shows Δ*R/R* measured at 150 K with pump fluences ranging from 0.46 to 4.6 mJ/cm^2^. The sharp initial positive peak of Δ*R/R* was observed for fluences of 0.46 mJ/cm^2^ and 1.15 mJ/cm^2^. At higher fluences, the dynamics became growth-like immediately after the arrival of the pump pulse. Figure 3(d) shows fluence-dependent Δ*R/R* at 300 K. At this temperature, “growth-like” dynamics was observed for all fluences, which is expected as VO_2_(*B*) is already deep into the metallic phase.

The three sets of data in each of Fig. 2(a–c) show the temperature dependence of *A, t*_*P*_, and *τ*_1_, for pump fluences of 1.15, 1.8 and 3.45 mJ/cm^2^ pump fluence. For pump fluences of 1.15 and 1.8 mJ/cm^2^ we see a similar trend — (1) the coefficient *A* going from positive in the low-temperature insulating phase, to negative in the high-temperature metallic phase, changing sign at ~170 K (at 1.15 mJ/cm^2^ pump fluence) and ~140 K (at 1.8 mJ/cm^2^ pump fluence), and (2) *t*_P_ abruptly increasing from sub-ps to ~10 ps at ~170 K (at 1.15 mJ/cm^2^ pump fluence) and ~140 K (at 1.8 mJ/cm^2^ pump fluence), and (3) *τ*_1_ exhibiting a broad peak near ~170 K (at 1.15 mJ/cm^2^ pump fluence) and ~140 K (at 1.8 mJ/cm^2^ pump fluence). On the other hand, for all temperatures at a pump fluence of 3.45 mJ/cm^2^, the coefficient *A* remains negative. The change in *A* from positive to negative, and the concomitant lengthening of *τ*_1_, could signify the melting of the insulating order in VO_2_(*B*), which we will discuss later in the context of photoinduced phase transition.

Figure 2(d–f) show the fluence-dependent plots of *A, t*_*P*_, and *τ*_1_ at a fixed temperature, and shows similar trends to the temperature-dependent plots. As the pump fluence is systematically increased while keeping the temperature constant at 150 K, Δ*R/R* changed from “relaxation-like” to “growth-like” at a “threshold” fluence, *F*_*th*_, of 1.5 mJ/cm^2^, as shown by the change of sign of *A* from positive to negative. There is also a concomitant lengthening of *τ*_1_ at the same value of *F*_*th*_. Also note that, as temperature is increased, *F*_*th*_ becomes lower — this behaviour is seen in both the temperature and fluence-dependent data. The value of *F*_*th*_ differs significantly between VO_2_(*M*) and VO_2_(*B*) — at room temperature, VO_2_(*M*) is completely insulating and requires *F*_*th*_ of 6‒7 mJ/cm^2^ but if we chose 150 K as the analogous temperature in VO_2_(*B*), the required *F*_*th*_ is only 1.5 mJ/cm^2^ 31.

**Figure 4:**
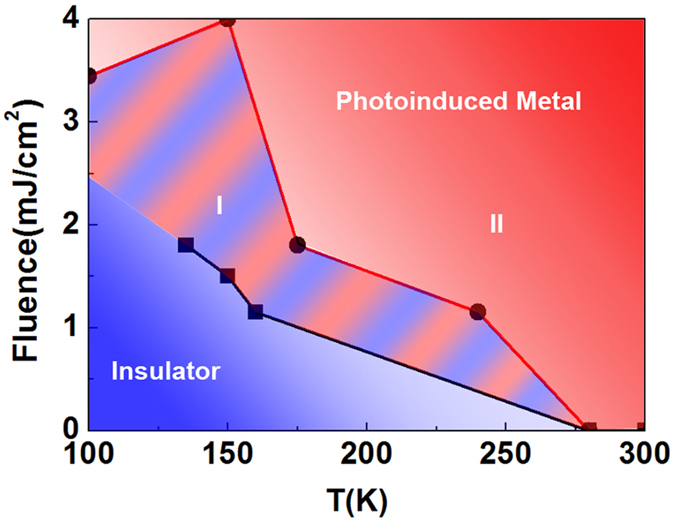
Summary of the dynamics study of VO_2_(*B*) as represented by the phase diagram. The red and the blue shaded regions represent the metallic state and the insulating states respectively. The metallic phase is further subdivided into two regions — (I) thermally induced exhibiting longer *τ*_1_ (light blue and red streaks) and (II) photoinduced (coloured red) where *τ*_1_ ≤ 3 ps.

The photoinduced MIT transition is illustrated by mapping the phase diagram of VO_2_(*B*) in non-equilibrium conditions [Fig. 4]. The phase boundaries are estimated from the values of *F*_*th*_ determined from fluence-dependent and temperature-dependent data (eg. *F*_*th*_ at 150 K is 1.5 mJ/cm^2^) where the coefficient *A* crosses from positive to negative. The metallic state is further subdivided into two regimes — I and II to represent the slow growth and the fast growth dynamics respectively. When in the photoinduced metallic state of VO_2_(*B*), we designate as regime I when *τ*_1_ > 3 ps, and regime II when *τ*_1_ ≤ 3 ps. This separation of regimes I and II is based on our data which indicates that for a fluence of 3.45 mJ/cm^2^, the system is in regime II and at this fluence, *τ*_1_ ≤ 3 ps.

We now discuss the physical origins of the different components. The fast relaxation time, *τ*_1_ in the insulating state of VO_2_(*B*) could arise from self-trapping of photoexcited carriers similar to the observations in VO_2_(*M*)^26^,^28^,^37^. On the other hand, the origin of *τ*_1_ in the ‘metallic-like’ state of VO_2_(*B*) is difficult to interpret. In the transition region going from the insulating to metallic state, whether as a function of temperature or fluence, excited carriers may be temporally trapped in metastable, transient energy states leading to a large *τ*_1_. The increase of relaxation time with increasing pump fluence has also been reported in systems like intercalated bilayer graphene — there the increase in the electron-phonon relaxation time was explained using the reabsorption of optical phonons by the carriers leading to a quasi-equilibrium state which slows the rate of hot electron cooling^38^.

In VO_2_(*M*), the growth dynamics in the photoinduced metallic phase is interpreted as a result of seeding of metallic nano-domains by light which eventually expands outwards into the insulating regions via dynamical and percolating growth^28^,^35^. A complete phase transition of photoexcited VO_2_(*M*) may take as long as 100 ps; beyond that, photoexcitation merely increases the film temperature^29^,^35^,^39^. The time scale of the growth dynamics in VO_2_(*B*), in regime I, falls in this range. Lattice heating due to the pump laser can account for the energy required to cross into metallic regime I, and in this scenario nucleation and growth of metallic phases are possible. However the laser energy is not sufficient for the system to reach metallic regime II. One can estimate the upper limit of increase in lattice temperature by considering that the entire pump energy has been converted to absorbed heat. The corresponding temperature change for fluence as large as 3.45 mJ/cm^2^ is only 32.5 K compared to the large transition width of 150 K (Supplementary information). This means that the pure lattice heating effects can drive the insulating state of VO_2_(*B*) to regime I in the phase diagram but not into regime II.

Further calculations at each of the temperature points show that the temperature rise, Δ*T* due to lattice heating is inversely proportional to the initial system temperature (Supplementary information). In Fig. 4, the width of regime I decreases with increasing temperature. This shape of the phase boundary in regime I agree with our calculations.

The photoinduced metallic phase in regime II can be understood by estimating the carrier density in the system. Assuming that one pump photon creates one free charge carrier, the density of the photogenerated carriers, Δ*n* can be estimated using the relation 
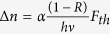
 where *R* = 0.11 is the reflectivity of light (800 nm) in the low-temperature insulating state, *v* is the corresponding frequency for a wavelength of 800 nm, and absorption coefficient α = 5.5 × 10^4^ cm^−1^ for VO_2_(*B*) film as obtained from the absorbance spectrum (Supplementary Information). At a pump fluence of 3.45 mJ/cm^2^, Δ*n* ~ 3.1 × 10^21^ cm^−3^. It is important to mention here that this value of photogenerated carrier density in VO_2_(*B*) is comparable to the free-carrier density in metallic VO_2_(*B*) (~10^22^ cm^−3^) obtained from Hall experiments at 300 K^23^. At such a large free-carrier concentration, the system cannot remain insulating until the system completely relaxes. This is six orders of magnitude larger than the carrier density in the insulating phase^23^.

## Discussion

In summary, the photoinduced effect on the various electronic phases of VO_2_(*B*) has been studied for the first time by ultrafast pump-probe technique. Transient reflectivity (Δ*R/R*) dynamics demonstrate that the insulating state has an initial sharp peak and decays within a few ps. The metallic state induced by lattice heating from the pump laser has a slower growth of Δ*R/R* and survives for a longer time becoming as long as several tens of ps. Finally, we discuss the possibility of a pure photoinduced phase transition in the regime which cannot be accounted by simple lattice heating. Our results should guide future time-resolved multi-color experiments on this system. Experiments at lower repetition rates will help to disentangle the lattice heating effects with that of a pure photoinduced phase transition.

## Methods

### Sample growth

50-nm VO_2_(*B*) thin films were fabricated by Pulsed Laser Deposition by ablating a commercial vanadium metallic target. The substrate chosen was specially fabricated Si with a buffer layer of 20 nm SrTiO_3_ (SiSTO). The growth of *B*-phase VO_2_ thin films was stabilized at a temperature of 500 °C and a fixed laser energy density of 2 J/cm^2^ and the oxygen pressure was kept between 5 × 10^−3^ to 7 × 10^−3^ Torr. The thickness of VO_2_(*B*) thin film used for the pump-probe study was ~50 nm. The detailed growth study has been discussed in an earlier publication^23^.

### OPOP

The transient reflectivity measurements in our pump-probe experiments were performed using a Ti:sapphire regenerative amplifier system (Coherent RegA with a repetition rate of 250 kHz). Sub-50 fs pulses at a center wavelength of 800 nm generated by the laser was used as a source of both pump and probe pulses. The pump and probe pulses were cross polarized. The beam diameter of the pump was set to ~50 μm while the probe beam was focused to a smaller diameter of 25 μm which ensured an excellent pump-probe overlap. The reflected probe beam was focused onto a photodiode detector which was connected to a lock-in amplifier where the photoinduced changes in reflectivity Δ*R/R* were recorded. Temperature and fluence dependent measurements were taken on the sample which was mounted on a continuous flow cryostat. For fluence dependent measurements, the probe power was kept constant while the pump power was steadily increased without any other changes in the beam parameters.

### UV-Vis

We performed absorbance spectrum measurements in the range 220‒900 nm with the help of commercial UV-Vis spectrophotometer (Jasco, Model V-650).

## Additional Information

**How to cite this article**: Lourembam, J. *et al*. Evidence for Photoinduced Insulator-to-Metal transition in *B*-phase vanadium dioxide. *Sci. Rep.* **6**, 25538; doi: 10.1038/srep25538 (2016).

## Supplementary Material

Supplementary Information
