# Supplementary material for: Evidence for Photoinduced Insulator-to-Metal transition in B-phase vanadium dioxide
Source: Sci Rep. 2016 May 9;6:25538. doi: 10.1038/srep25538 (PMC4860617; doi:10.1038/srep25538)
Supplement: Supplementary Information [file srep25538-s1.doc]

**Supplementary Information**

**Evidence for Photoinduced Insulator-to-Metal transition in *B*-phase vanadium dioxide**

James Lourembam ^1^†, Amar Srivastava^2,3^, Chan La-o-vorakiat^4,5,6^, Cheng Liang^1^, T.Venkatesan^2,3,7,8,9^, Elbert E. M. Chia ^1^

*^1^Division of Physics and Applied Physics, School of Physical and Mathematical Sciences, Nanyang Technological University, Singapore 637371, Singapore.*

*^2^NUSNNI-Nanocore, National University of Singapore, Singapore 117411, Singapore.*

*^3^Department of Physics, National University of Singapore, Singapore 117542, Singapore.*

*^4^Nanoscience and Nanotechnology Graduate Program, King Mongkut’s University of Technology Thonburi (KMUTT), 10140, Thailand.*

*^5^Faculty of Science, King Mongkut’s University of Technology Thonburi (KMUTT), 10140, Thailand.*

*^6^Theoretical and Computational Science Center (TaCS), Faculty of Science, King Mongkut’s University of Technology Thonburi (KMUTT), 10140, Thailand.*

*^7^Department of Electrical and Computer Engineering, National University of Singapore, Singapore 117576, Singapore.*

*^8^Department of Materials Sciences and Engineering, National University of Singapore, Singapore 117576, Singapore.*

*^9^Department of Integrated Sciences and Engineering, National University of Singapore, Singapore 117456, Singapore.*

†Present address: Data Storage Institute, 2 Fusionopolis Way, Singapore 138634, Singapore

^*^elbertchia@ntu.edu.sg, venky@nus.edu.sg

1. BI-EXPONENTIAL FITTINGS OF TRANSIENT REFLECTIVITES


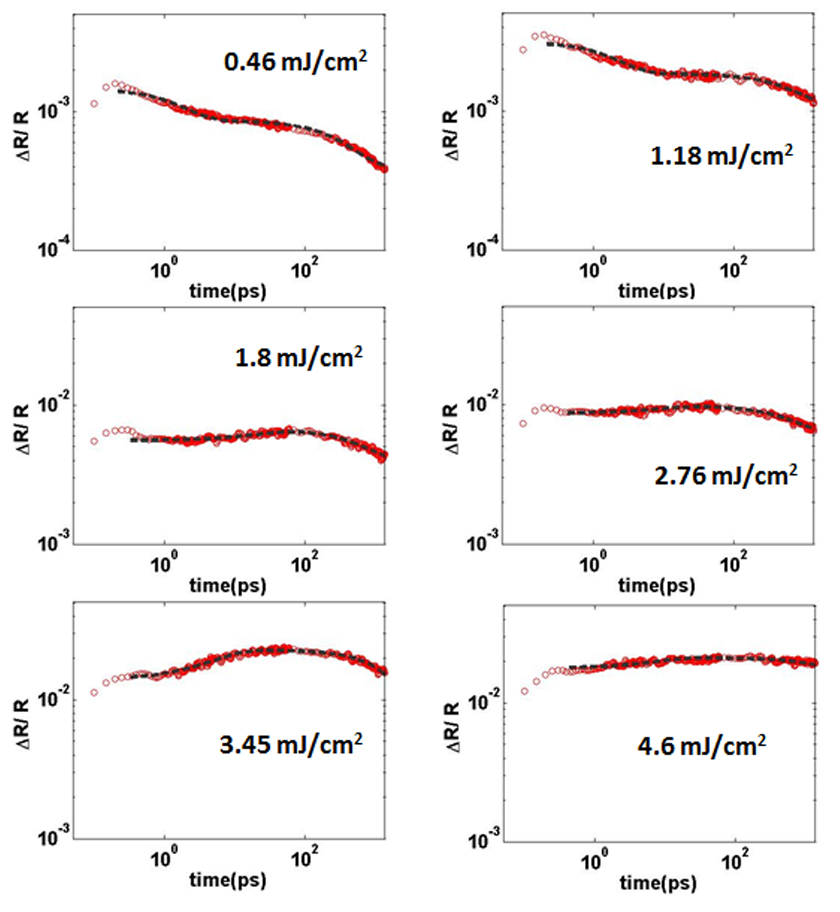


Supplementary figure 1: Plot of *ΔR/R* vs. time of VO_2_(*B*) at 150 K shown along with the bi-exponential fittings for different fluences.


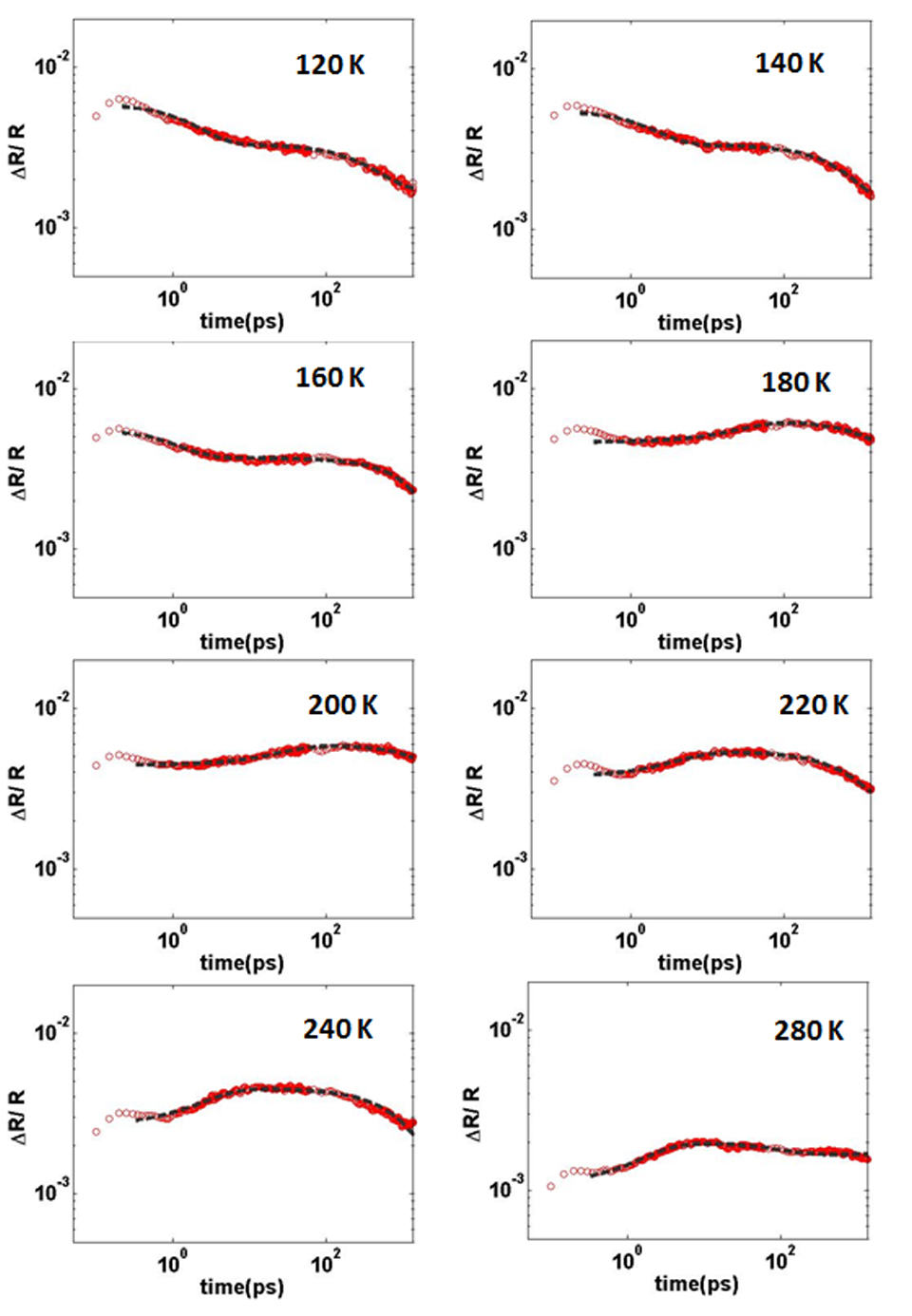


Supplementary figure 2: Plot of *ΔR/R* vs. time of VO_2_(*B*) at 1.15 mJ/cm^2^ shown along with the bi-exponential fittings for different temperatures.


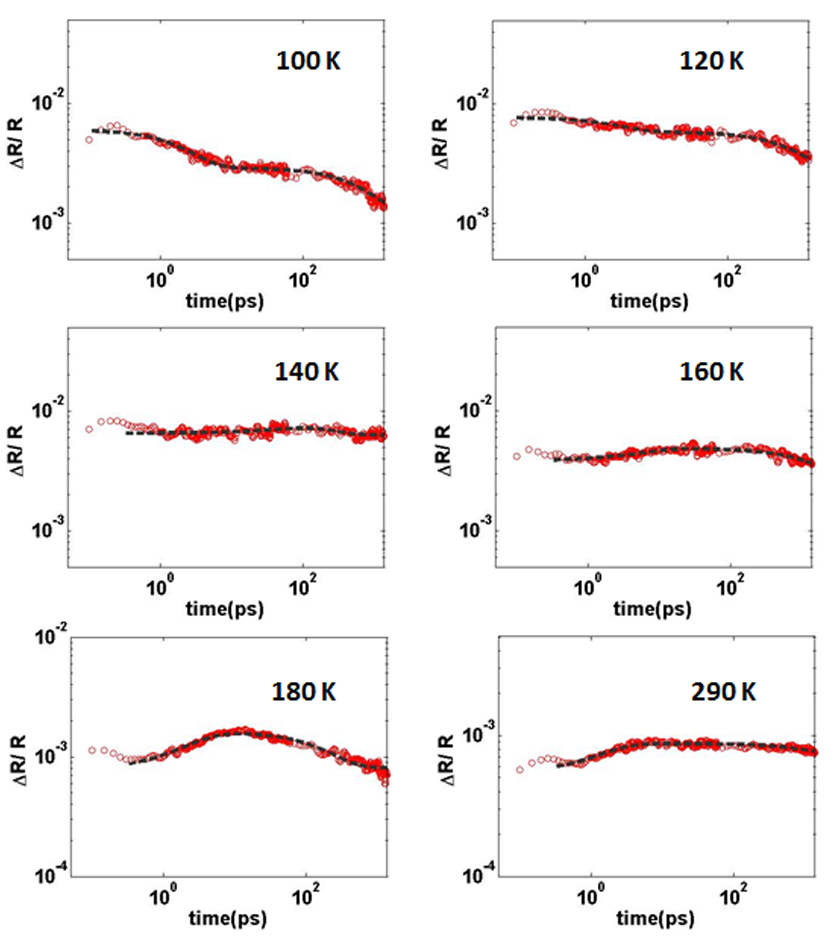


Supplementary figure 3: Plot of *ΔR/R* vs. time of VO_2_(*B*) at 1.8 mJ/cm^2^ shown along with the bi-exponential fittings for different temperatures.

1. FITTING PARAMETERS


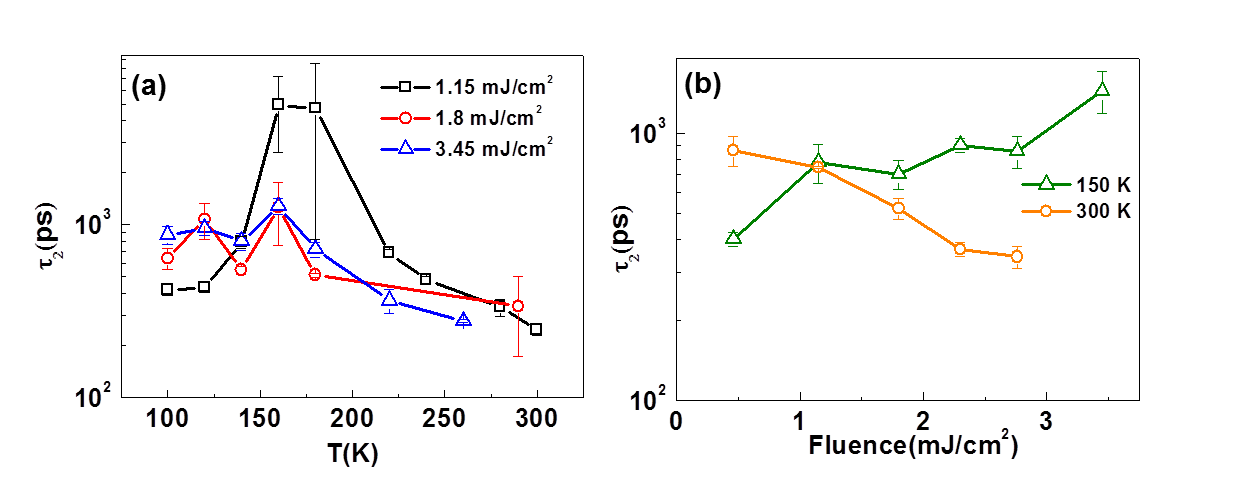


Supplementary figure 4: Plot of
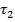
 as function of (a) temperature and (b) fluence for VO_2_(*B*) obtained by fitting the transient reflectivity curves by an bi-exponential model.


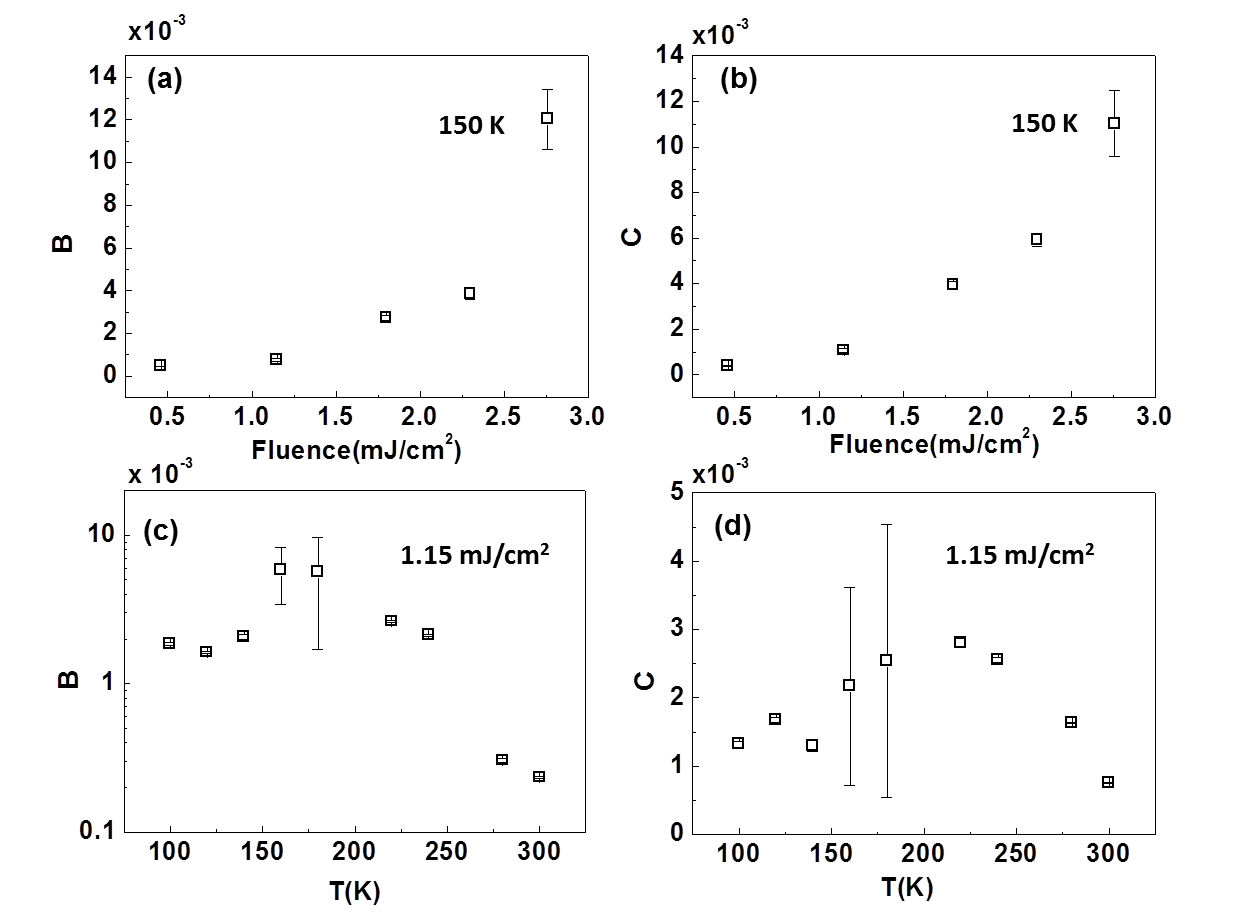


Supplementary figure 5: Plot of B and C as a function of fluence at 150 K ((a) and (b)) and as a function of temperature at a fluence of 1.15 mJ/cm^2^ ((c) and (d)).

1. CALCULATION OF FILM TEMPERATURE

The temperature increase in the photoexcited volume of the film due to the pump excitation is calculated from the formula,
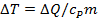
, where
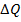
 is the heat absorbed,
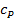
 is the specific heat, and *m* is the mass of the excited volume. The low temperature specific heat of VO_2_(*B*) was assumed to be the same as that of low temperature VO_2_(*M*1) which is given by 0.068 x 10^–4^ *T*^3^ cal K^–1^ mol^–1^ for *T* ≤ 25 K.[^1^](#_ENREF_1) The density of VO_2_(*B*) is 4.031 g cm^−3^.[^2^](#_ENREF_2) The amount of heat absorbed per volume is the product of fluence and optical penetration depth.

| Initial temperature, *T*_i_ | 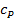 (J K^–1^ cm^–3^) | Fluence (mJ/cm^2^) | Temperature increase, Δ*T* |
| --- | --- | --- | --- |
| 150 K | 1.448 | 0.46 | 4.3 K |
| 150 K | 1.448 | 1.15 | 10.8 K |
| 150 K | 1.448 | 1.8 | 17 K |
| 150 K | 1.448 | 2.3 | 21.7 K |
| 150 K | 1.448 | 2.76 | 26 K |
| 150 K | 1.448 | 3.45 | 32.5 K |

Supplementary table I: Calculated increase in lattice temperature of VO_2_(*B*) for various fluences. The initial temperature or the substrate temperature in this case is 150 K.


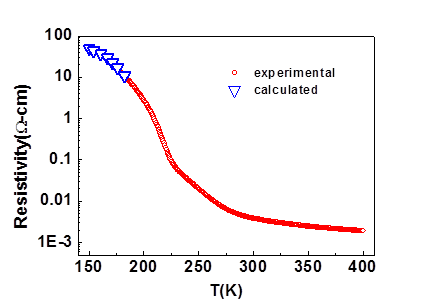


Supplementary figure 6: Temperature dependent resistivity curve of VO_2_(*B*) reproduced from Lourembam *et al*.[^3^](#_ENREF_3) The red symbol curve is experimentally determined from four point method. The blue symbols represent the calculated resistivity if only the lattice heating due to the pump laser is considered. The symbols represent the following fluences— (0.46, 1.15, 1.8, 2.3, 2.76, 3.45) mJ/cm^2^.

| Initial temperature, *T*_i_ | 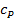 (J K^–1^ cm^–3^) | Fluence (mJ/cm^2^) | Temperature increase, Δ*T* |
| --- | --- | --- | --- |
| 100 K | 0.43 | 3.45 | 109.7 K |
| 120 K | 0.74 | 3.45 | 63.5 K |
| 140 K | 1.18 | 3.45 | 40 K |
| 160 K | 1.76 | 3.45 | 26.8 K |
| 180 K | 2.5 | 3.45 | 18.8 K |
| 220 K | 4.57 | 3.45 | 10.3 K |
| 300 K | 11.58 | 3.45 | 4 K |

Supplementary table II: Calculated increase in lattice temperature of VO_2_(*B*) for 3.45 mJ/cm^2^.

1. ABSORBANCE SPECTRA

Supplementary figure 8: Absorbance spectra of the VO_2_(*B*) thin film determined after subtracting the absorbance spectra of the substrate measured using a UV-Vis spectrophotometer at 300 K.

1 McWhan, D. B. *et al.* Heat Capacity of Vanadium Oxides at Low Temperature. *Phys. Rev. B* **7**, 326-332, (1973).

2 Leroux, C., Nihoul, G. & Tendeloo, G. V. From VO_2_(*B*) to VO_2_(*R*): Theoretical structures of VO_2_ polymorphs and *in situ* electron microscopy. *Phys. Rev. B* **57**, 5111, (1998).

3 Lourembam, J. *et al.* New insights into the electronic phase diagram of a novel vanadium dioxide polymorph: A terahertz spectroscopy study. *Sci. Rep.* **5**, 9182; 10.1038/srep09182 (2015).
